# Preoperative hematocrit levels and postoperative mortality in patients undergoing craniotomy for brain tumors

**DOI:** 10.3389/fonc.2023.1246220

**Published:** 2023-10-17

**Authors:** Yangchun Xiao, Xin Cheng, Lu Jia, Yixin Tian, Jialing He, Miao He, Lvlin Chen, Pengfei Hao, Tiangui Li, Weelic Chong, Yang Hai, Chao You, Liyuan Peng, Fang Fang, Yu Zhang

**Affiliations:** ^1^ Department of Neurosurgery, Clinical Medical College and Affiliated Hospital of Chengdu University, Chengdu, Sichuan, China; ^2^ Department of Neurosurgery, West China Hospital, Sichuan University, Chengdu, Sichuan, China; ^3^ Department of Neurosurgery, Shanxi Provincial People’s Hospital, Taiyuan, Shanxi, China; ^4^ Department of Anesthesia, Clinical Medical College and Affiliated Hospital of Chengdu University, Chengdu, Sichuan, China; ^5^ Department of Critical Care Medicine, Clinical Medical College and Affiliated Hospital of Chengdu University, Chengdu, Sichuan, China; ^6^ Department of Neurosurgery, Longquan Hospital, Chengdu, Sichuan, China; ^7^ Department of Medical Oncology, Thomas Jefferson University, Philadelphia, PA, United States; ^8^ Sidney Kimmel Medical College, Thomas Jefferson University, Philadelphia, PA, United States; ^9^ Center for Evidence Based Medical, Clinical Medical College and Affiliated Hospital of Chengdu University, Chengdu, Sichuan, China

**Keywords:** hematocrit, anemia, polycythemia, mortality, brain tumor, craniotomy

## Abstract

**Background:**

Abnormal hematocrit values, including anemia and polycythemia, are common in patients undergoing craniotomy, but the extent to which preoperative anemia or polycythemia independently increases the risk of mortality is unclear. This retrospective cohort study aimed to examine the association between preoperative anemia and polycythemia and postoperative mortality in patients who underwent craniotomy for brain tumor resection.

**Methods:**

We retrospectively analyzed data from 12,170 patients diagnosed with a brain tumor who underwent cranial surgery at West China Hospital between January 2011 and March 2021. The preoperative hematocrit value was defined as the last hematocrit value within 7 days before the operation, and patients were grouped according to the severity of their anemia or polycythemia. We assessed the primary outcome of 30-day postoperative mortality using logistic regression analysis adjusted for potential confounding factors.

**Results:**

Multivariable logistic regression analysis reported that the 30-day mortality risk was raised with increasing severity of both anemia and polycythemia. Odds ratios for mild, moderate, and severe anemia were 1.12 (95% CI: 0.79–1.60), 1.66 (95% CI: 1.06–2.58), and 2.24 (95% CI: 0.99–5.06), respectively. Odds ratios for mild, moderate, and severe polycythemia were 1.40 (95% CI: 0.95–2.07), 2.81 (95% CI: 1.32–5.99), and 14.32 (95% CI: 3.84–53.44), respectively.

**Conclusions:**

This study demonstrated that moderate to severe anemia and polycythemia are independently associated with increased postoperative mortality in patients undergoing craniotomy for brain tumor resection. These findings underscore the importance of identifying and managing abnormal hematocrit values before craniotomy surgery.

## Introduction

Preoperative abnormal hematocrit values, including anemia and polycythemia, are modifiable factors that are highly prevalent, occurring in 20% to 40% of surgical patients (1, 2). Substantial evidence has shown that both anemia and polycythemia have prognostic implications for the general population as well as for patients undergoing cardiac and noncardiac surgeries (1–5).

Although studies based on the National Surgical Quality Improvement Program (NSQIP) have shown that anemia increases the mortality risk in patients undergoing craniotomy (6, 7), it remains unclear whether mild anemia is also associated with an increased risk of mortality. This question is critical since the ideal transfusion trigger has yet to be established (8, 9). Moreover, available data do not account for the impact of preexisting comorbidities on this association. Consequently, it is unclear whether the effects of anemia on outcomes are directly caused by anemia or are a result of its association with other risk factors commonly present in patients with anemia.

Polycythemia has been linked to an increased chance of cardiovascular disease (10). In the same way, preoperative polycythemia has been found to be associated with a higher risk of postoperative mortality in major surgery (2, 11, 12). Neurosurgical patients are particularly susceptible to polycythemia because the high red blood cell count can lead to blood clots in the brain and consequently an ischemic stroke. During surgery, the brain requires a continuous supply of oxygen, and therefore individuals with polycythemia are at an elevated risk of hypoxia due to the reduced flow of oxygen-rich blood to the brain. Similar associations have been reported in emergency conditions such as hemorrhagic and ischemic stroke (13–16). However, currently, there is no research available investigating the relationship between polycythemia and postoperative outcomes in the context of craniotomy.

Given that anemia and polycythemia are detectable in routine preoperative blood tests and potentially treatable, it is essential to understand their effects on perioperative outcomes. Therefore, we conducted a comprehensive study to investigate the association between preoperative anemia and polycythemia and postoperative mortality in patients undergoing craniotomy for a brain tumor. Specifically, we evaluated the impact of comorbidities and other risk factors, as assessed by the Charlson Comorbidity Index (17), on the relationship between abnormal hematocrit values and postoperative outcomes

## Patients and methods

### Study design

In this retrospective, single-center cohort study, we identified patients diagnosed with a brain tumor who underwent cranial surgery. All patients were collected from West China Hospital (between January 2011 to March 2021). Data acquisition for this study was extracted from electronic medical records. Patients’ survival information was collected from the Household Registration Administration System of the People’s Republic of China, in which residents’ mortality records were updated at the time of death. Approval was obtained from the institutional review board of the ethics committee of [BLINDED FOR REVIEW] on 29 April 2022. Informed consent was exempted because our study was a clinical audit (The ethics committee of West China Hospital. Sichuan University No. 2022-705).

### Patient selection

We identified patients’ primary diagnoses of brain tumors according to the International Classification of Diseases, tenth revision (ICD-10) codes. All patients who underwent craniotomies were included in the study.

Patients were excluded according to the following criteria (1): patients whose preoperative hematocrit levels were lacking (2); patients with multiple operations in the same hospital stay or reoperations within 30 days; and (3) patients whose personal identification number was not found in the electronic medical records because we extracted patients’ survival information by personal identification number from the Household Registration Administration System.

### Clinical characteristics

In this study, the following demographic characteristics are collected: demographics including age, sex, current smoking status, and alcohol consumption; preoperative comorbidities including hypertension, diabetes, chronic liver disease, coronary artery disease, American Society of Anesthesiologists (ASA) class divided into grades I–II together and III–IV together, Charlson Comorbidity Index, primary tumor diagnosis, and histopathology; the last systolic blood pressure before the operation within 3 days; the last preoperative laboratory test before the operation within 7 days; preoperative anticoagulant/antiplatelet drugs; preoperative transfusion; intraoperative features including surgical duration and intraoperative blood loss; and grade of resection including gross total resection and subtotal resection.

### Exposure

The preoperative hematocrit value was defined as the last hematocrit value within 7 days before the operation. According to the World Health Organization classification, anemia is defined as a hematocrit less than 39.0% for men and less than 36.0% for women (18). Based on previous studies, preoperative anemia is defined as a hematocrit value of less than 39.0% (5).

We first performed restricted cubic splines to show the hazard rate of hematocrit and mortality. According to restricted cubic splines results, we detected an increase in the hazard rate of postoperative mortality when hematocrit is less than 39.1% and more than 45%. Therefore, patients with hematocrit between 39.1% and 45% were defined as a normal hematocrit group. The polycythemia group is defined as having a hematocrit value of more than 45%. Furthermore, we divided the anemia group into three groups: severe anemia (≤30.0%), moderate anemia (30.1%–35.0%), and mild anemia (35.1%–39.0%). The polycythemia group was divided into three groups: mild polycythemia (45.1%–50%), moderate polycythemia (50.1%–55.0%), and severe polycythemia (>55.0%).

### Outcome measures

The primary outcome of this study was 30-day mortality, defined as all mortality within 30 days. We also assessed long-term mortality at one year. Patients’ mortality information was collected from the Household Registration Administration System of the People’s Republic of China, in which residents’ mortality records were updated at the time of death as required by law. All patients’ mortality information was collected on 1 April 2021.

### Statistical analysis

The R version 4.21 (R package for Statistical Computing) was used to perform all statistical analyses. Values are expressed as mean with standard deviation for continuous variables and as frequency counts with percentages for categorical variables. Chi-squared tests were conducted for categorical variables, and Wilcoxon ranks sum test (Kruskal–Wallis test for >2 categories) for continuous variables. A multiple imputation method was performed for missing data (19).

We performed a univariable logistic regression analysis to assess the association of demographic variables and clinical characteristics with mortality, based on prior studies and literature. The study considered several potential confounding variables, including age, sex, current smoking status, alcohol consumption, hypertension, diabetes, chronic liver disease, coronary artery disease, Charlson Comorbidity Index (20), ASA class, primary tumor diagnosis, histopathology (21–23), systolic blood pressure, platelets, neutrophil count, serum albumin, lymphocyte count, blood glucose, international normalized ratio, prothrombin time, activated partial thromboplastin time, and preoperative anticoagulant/platelet drugs. Variables with a *p*-value less than 0.10 in the univariate logistic regression analyses were selected as confounding variables in the multivariable logistic regression. The multivariable logistic regression was performed with the backward stepwise method to assess the association of preoperative anemia with 30-day mortality. A two-sided *p*-value less than 0.05 was considered statistical significance. We used variance inflation factor (VIF) analysis to assess the degree of multicollinearity between the covariates in the multivariable logistic regression. VIF less than 10 were considered to have no collinearity. Furthermore, we assessed the pattern and magnitude of associations between hematocrit and mortality with restricted cubic splines performed by a logistic regression model.

The incidence of patients surviving for 30 days after neurosurgical treatment was examined by the Kaplan–Meier analyses, and differences were compared by the log-rank test between anemia, normal hematocrit, and polycythemia. We performed multivariate Cox proportional hazard regression models to assess the independent factors for overall survival, expressed as an adjusted hazard ratio (HR) and 95% confidence interval (CI).

We conducted subgroup analyses separately to examine the modifying effect by age (≤65 years and >65 years), sex (male and female), smoking, alcohol, hypertension, diabetes, chronic liver disease, Charlson Comorbidity Index, coronary artery disease, ASA, systolic blood pressure, preoperative transfusion, and preoperative anticoagulant platelet between anemia, normal hematocrit, and high hematocrit groups.

## Results

### Clinical characteristics

**Figure 1:**
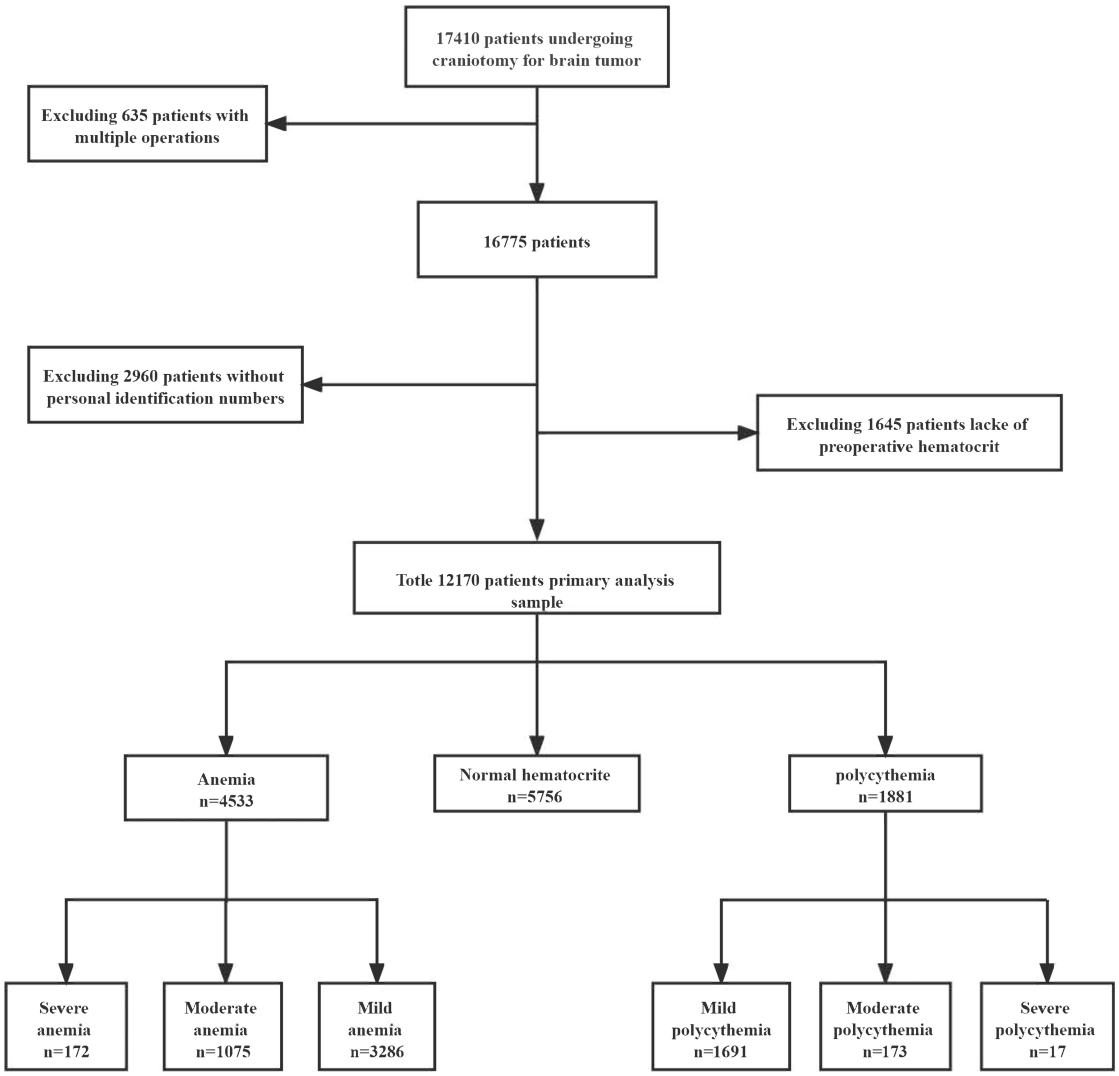
Flow chart of enrollment.

We identified 17,410 adult patients diagnosed with brain tumors who underwent craniotomies. We excluded patients from this study (1,944 (11.2%) patients without preoperative hematocrit, 635 (3.6%) patients with multiple operations, and 2,960 (17%) patients without personal identification numbers) (**Figure 1**). Finally, a total of 12,170 patients were included in this study. In this study, 172 (1.4%) patients were diagnosed with severe anemia, 1,075 (8.8%) patients with moderate anemia, 3,286 (27.0%) patients with mild anemia, 1,691 (13.9%) patients with mild polycythemia, 173 (1.4%) patients with moderate polycythemia, and 17 (0.14%) patients with severe polycythemia (**Table 1**). The overall 30-day mortality in this study was 221 (1.7%). Patients with anemia were more likely to have more women, to smoke less, to drink less alcohol, to have more benign tumors, and to have low systolic blood pressure. Patients with polycythemia were more likely to have fewer women, to smoke more, to drink more alcohol, to have hypertension, to have more malignant tumors, and to have high systolic blood pressure.

**Table 1:** Baseline characteristics of the patients.

Characteristics	Low hematocrit			Normal hematocrit	High hematocrit			*p*-value^*^	*p*-value^#^
	Severe (n = 172)	Moderate (n = 1,075)	Mild (n = 3,286)	n = 5,756	Mild (n = 1,691)	Moderate (n = 173)	Severe (n = 17)		
Demographics									
Age (mean (SD))	47.91 (15.19)	45.88 (18.17)	47.48 (17.34)	48.05 (16.16)	45.89 (14.31)	40.42 (13.51)	48.59 (12.77)	0.478	<0.001
Female (*n* (%))	130 (75.6)	814 (75.7)	2,557 (77.8)	3,035 (52.7)	169 (10.0)	3 (1.7)	0 (0.0)	<0.001	<0.001
Smoking (*n* (%))	8 (4.7)	48 (4.5)	139 (4.2)	613 (10.6)	365 (21.6)	42 (24.3)	4 (23.5)	<0.001	<0.001
Alcohol (*n* (%))	18 (10.5)	92 (8.6)	235 (7.2)	886 (15.4)	472 (27.9)	52 (30.1)	5 (29.4)	<0.001	<0.001
Medical history (n (%))									
Hypertension	25 (14.5)	177 (16.5)	477 (14.5)	913 (15.9)	228 (13.5)	22 (12.7)	2 (11.8)	0.23	0.011
Diabetes	14 (8.1)	78 (7.3)	239 (7.3)	427 (7.4)	104 (6.2)	8 (4.6)	2 (11.8)	0.852	0.052
Chronic liver disease	12 (7.0)	54 (5.0)	140 (4.3)	294 (5.1)	102 (6.0)	8 (4.6)	0 (0.0)	0.203	0.236
Coronary artery disease	2 (1.2)	20 (1.9)	37 (1.1)	65 (1.1)	11 (0.7)	0 (0.0)	0 (0.0)	0.481	0.053
ASA class (n (%))								0.103	0.713
I–II	109 (63.4)	597 (55.5)	1,919 (58.4)	3,447 (59.9)	992 (58.7)	106 (61.3)	10 (58.8)		
III–V	63 (36.6)	478 (44.5)	1,364 (41.5)	2,307 (40.1)	698 (41.3)	67 (38.7)	7 (41.2)		
CCI ≥ 1 (*n* (%))	77 (44.8)	441 (41.0)	1,292 (39.3)	2,460 (42.7)	874 (51.7)	87 (50.3)	5 (29.4)	0.004	<0.001
Primary diagnosis (n (%))								<0.001	<0.001
Schwannoma	8 (4.7)	54 (5.0)	171 (5.2)	321 (5.6)	111 (6.6)	8 (4.6)	1 (5.9)		
Meningioma	34 (19.8)	273 (25.4)	1,023 (31.1)	1,618 (28.1)	401 (23.7)	38 (22.0)	3 (17.6)		
Craniopharyngioma	34 (19.8)	273 (25.4)	1,023 (31.1)	1,618 (28.1)	401 (23.7)	38 (22.0)	3 (17.6)		
Glioma WHO grades I–II	24 (14.0)	155 (14.4)	458 (13.9)	886 (15.4)	300 (17.7)	37 (21.4)	10 (58.8)		
Glioma WHO grades III–IV	23 (13.4)	211 (19.6)	654 (19.9)	1,328 (23.1)	552 (32.6)	66 (38.2)	2 (11.8)		
Metastasis	22 (12.8)	56 (5.2)	145 (4.4)	272 (4.7)	77 (4.6)	6 (3.5)	0 (0.0)		
Other	52 (30.2)	278 (25.9)	741 (22.6)	1,188 (20.6)	227 (13.4)	18 (10.4)	1 (5.9)		
Histopathology (n (%))								<0.001	<0.001
Benign	89 (51.7)	588 (54.7)	1,880 (57.2)	3,029 (52.6)	702 (41.5)	66 (38.2)	5 (29.4)		
Borderline	26 (15.1)	150 (14.0)	394 (12.0)	703 (12.2)	193 (11.4)	26 (15.0)	9 (52.9)		
Malignant	54 (31.4)	308 (28.7)	924 (28.1)	1,843 (32.0)	737 (43.6)	79 (45.7)	2 (11.8)		
Uncertain	3 (1.7)	29 (2.7)	88 (2.7)	181 (3.1)	59 (3.5)	2 (1.2)	1 (5.9)		
Systolic blood pressure (mean (SD))	122 (18.9)	121 (18.7)	123 (17.5)	125 (16.6)	127 (15.2)	128 (15.7)	126 (21.2)	<0.001	<0.001
Preoperative laboratory (mean (SD))									
Platelet (10^9^/L)	208 (106.9)	193 (86.6)	189 (69.7)	192 (65.2)	187 (57.5)	190 (56.1)	166 (50.2)	0.473	0.005
Neutrophil count (10^9^/L)	5.94 (4.07)	4.40 (2.97)	4.05 (2.36)	4.43 (2.54)	4.94 (2.70)	5.17 (2.71)	5.49 (2.54)	<0.001	<0.001
Serum albumin (g/L)	37.91 (5.37)	40.76 (4.06)	42.10 (3.47)	43.08 (3.40)	44.26 (3.39)	45.18 (3.78)	43.11 (3.68)	<0.001	<0.001
Lymphocyte count (10^9^/L)	1.52 (1.80)	1.64 (0.88)	1.75 (0.79)	1.82 (0.71)	1.83 (0.66)	1.97 (0.62)	1.56 (0.64)	<0.001	0.232
Blood glucose (mmol/L)	5.87 (1.89)	5.42 (1.51)	5.34 (1.46)	5.42 (1.57)	5.46 (1.55)	5.38 (1.74)	5.38 (1.71)	0.189	0.39
International normalized ratio	1.03 (0.08)	1.00 (0.08)	0.98 (0.08)	0.97 (0.07)	0.97 (0.07)	0.97 (0.08)	1.01 (0.13)	<0.001	0.428
Prothrombin time (s)	11.70 (0.93)	11.36 (0.92)	11.16 (0.83)	11.06 (0.83)	11.04 (0.83)	11.08 (0.97)	11.31 (1.51)	<0.001	0.612
APTT (s)	28.54 (4.88)	28.32 (6.61)	27.63 (3.82)	27.25 (3.77)	27.34 (3.68)	28.25 (3.92)	29.16 (3.56)	<0.001	0.059
Preoperative transfusion (n (%))	19 (11.0)	11 (1.0)	6 (0.2)	5 (0.1)	0 (0.0)	0 (0.0)	0 (0.0)	<0.001	0.448
Preoperative anti-coagulant/platelet drugs (n (%))	27 (15.7)	77 (7.2)	125 (3.8)	168 (2.9)	46 (2.7)	4 (2.3)	1 (5.9)	<0.001	0.698
Intraoperative features (mean (SD))									
Surgical duration (h)	3.50 (1.60)	3.41 (1.65)	3.54 (1.81)	3.64 (1.79)	3.84 (1.82)	3.81 (1.68)	4.00 (2.52)	<0.001	<0.001
Blood loss (mL)	450 (650)	301 (497)	277 (450)	282(462)	327 (487)	321 (428)	296 (249)	0.458	0.001
Grade of resection (n (%))									
Subtotal resection	36 (20.9)	248 (23.1)	707 (21.5)	1,442 (25.1)	579 (34.2)	75 (43.4)	9 (52.9)	<0.001	<0.001
Gross total resection	136 (79.1)	827 (76.9)	2,579 (78.5)	4,314 (74.9)	1,112 (65.8)	98 (56.6)	8 (47.1)		

Number of patients with data missing in the medical record (%): ASA 6 (0.05), SBP 301 (2.47), platelet 1,699 (13.96), International normalized ratio 761 (6.25), prothrombin time 480 (3.94), activated partial thromboplastin time 424(3.48), neutrophil count 83(0.68), Serum albumin 195 (1.60), blood glucose 209 (1.72), lymphocyte count 237 (1.95), surgical duration 153 (1.26), and blood loss 1,194 (9.81).

ASA, American Society of Anesthesiologists; CCI, Charlson Comorbidity Index; WHO, World Health Organization; APTT. activated partial thromboplastin time.

^*^Compared low hematocrit with normal hematocrit.

^#^Compared high hematocrit with normal hematocrit.

### Restricted cubic splines

**Figure 2:**
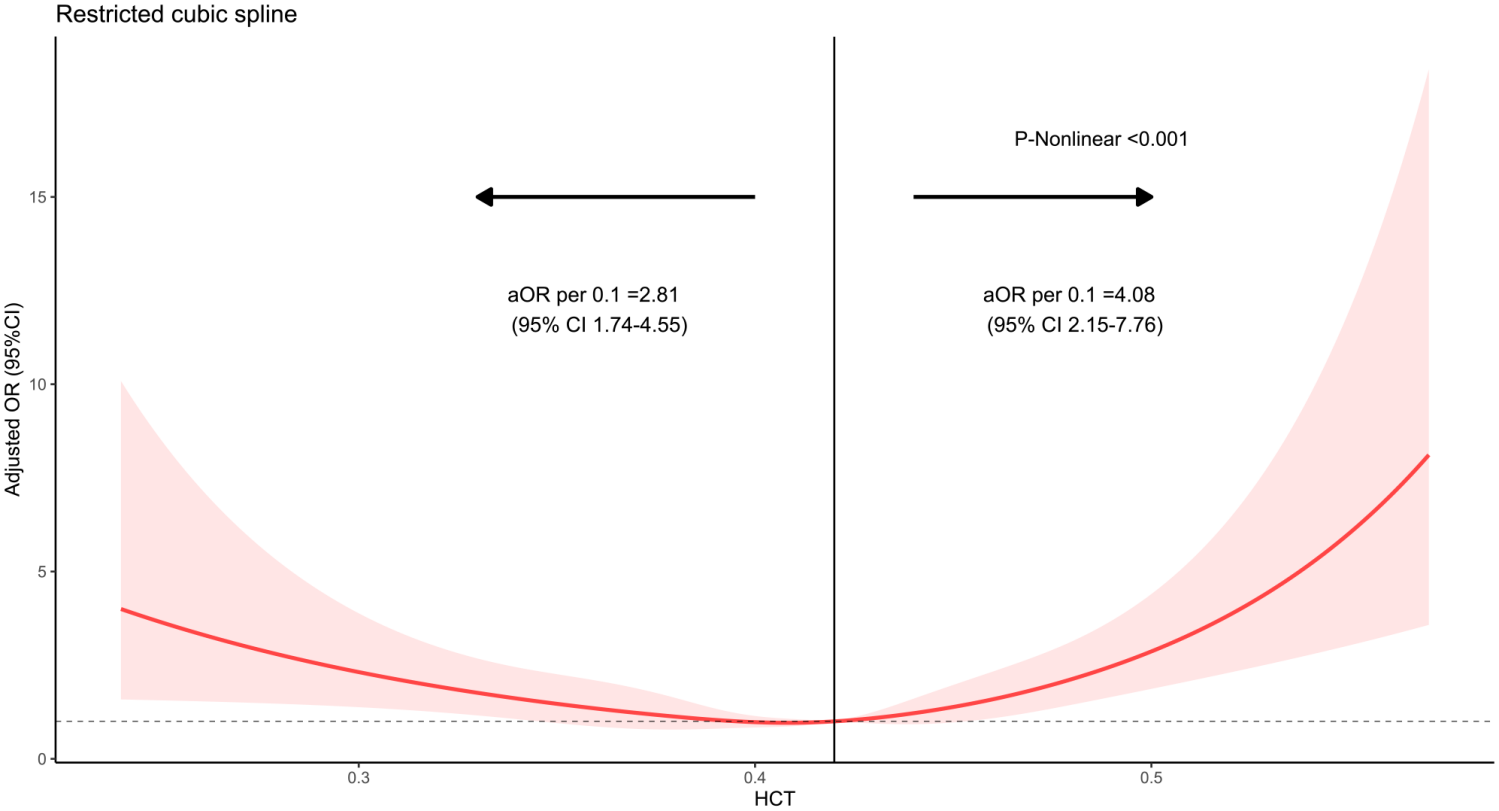
Restricted cubic spline of hematocrit with 30-day mortality in patients undergoing craniotomy. The *x*-axis shows hematocrit, and the *y*-axis shows the odds ratio of postoperative 30-day mortality.

We observed a U-shaped association between preoperative hematocrit and the probability of 30-day mortality in restricted cubic splines. The probability of 30-day mortality seemed lowest when preoperative hematocrit reached approximately 41% (**Figure 2**).

### Multivariable logistic regression analysis

**Figure 3:**
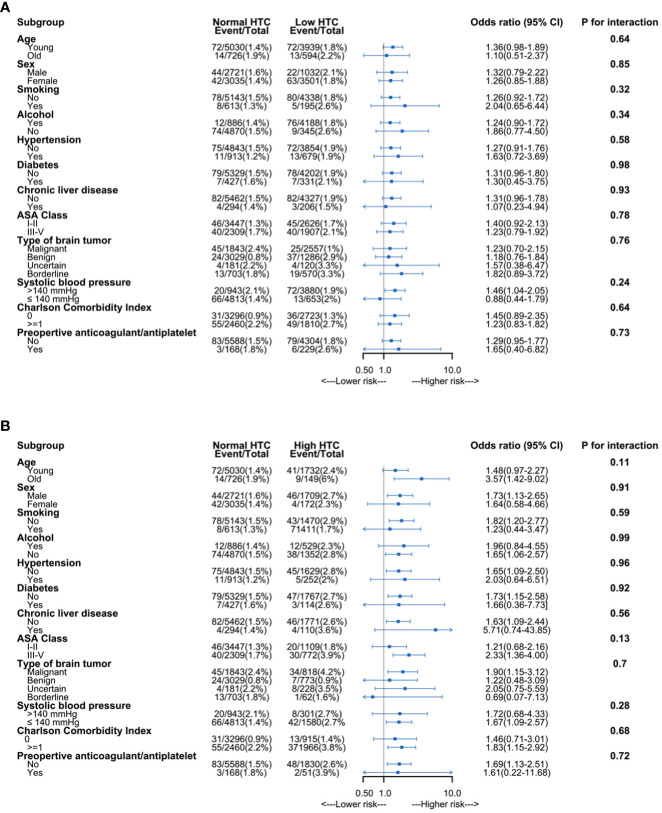
Subgroup analysis of association between hematocrit and 30-day mortality. **(A)** Association between anemia and 30-day mortality. **(B)** Association between polycythemia and 30-day mortality. HCT, hematocrit; ASA, American Society of Anesthesiologists.

To assess the 30-day mortality, we performed univariable and multivariable logistic regressions (**Supplementary Table S1**). We performed collinearity diagnostics with VIF. Significant collinearity was observed between the primary diagnosis and histopathology. We excluded histopathology from confounders. All the VIF values of selected confounders were separately lower than 10, and the mean VIF was 1.41. On the univariable logistic regression analysis, both anemia and polycythemia of increased severity showed an increased risk of 30-day mortality. The odds ratios of hematocrit and 30-day mortality still significantly increased risk in the multivariable logistic regression analysis. The odds ratios were 2.28 (95% CI: 1.02–5.11) for anemia and 2.27 (95% CI: 1.01–5.10) for polycythemia. The odds ratios were 2.24 (95% CI: 0.99–5.06) in patients with severe anemia, 1.66 (95% CI: 1.06–2.58) in patients with moderate anemia, 1.12 (95% CI: 0.79–1.60) in patients with mild anemia, 1.40 (95% CI: 0.95–2.07) in patients with mild polycythemia, 2.81 (95% CI: 1.32–5.99) in patients with moderate polycythemia, and 14.32 (95% CI: 3.84–53.44) in patients with severe polycythemia compared to normal hematocrit (**Table 2**). Variables with a *p <*0.10 on the univariable logistic regression analysis were selected as potential variables in the multivariable logistic regression. These variables included age, Charlson Comorbidity Index, coronary artery disease, ASA class, primary tumor diagnosis, neutrophil count, and blood glucose (**Table 3**). No significant interactions were present in subgroup analyses between anemia, normal hematocrit, and polycythemia (**Figure 3**).

**Table 2:** Unadjusted and adjusted association between hematocrit and 30-day mortality.

Characteristics	Hematocrit	Events/Total (n (%))	Unadjusted OR (95% CI)	Logistic regression adjustment (OR (95% CI))^*^
Severe anemia	≤0.3	7/172 (4.1%)	2.80 (1.27–6.14)	2.24 (0.99–5.06)
Moderate anemia	0.301–0.35	27/1,075 (2.5%)	1.70 (1.10–2.63)	1.66 (1.06–2.58)
Mild anemia	0.351–0.39	51/3,286 (1.6%)	1.04 (0.73–1.47)	1.12 (0.79–1.60)
Normal hematocrit	0.391–0.45	86/5,756 (1.5%)	1 (Reference)	1 (Reference)
Mild polycythemia	0.451–0.5	39/1,691 (2.3%)	1.56 (1.06–2.28)	1.40 (0.95–2.07)
Moderate polycythemia	0.501–0.55	8/173 (4.6%)	3.20 (1.52–6.71)	2.81 (1.32–5.99)
Severe polycythemia	>0.55	3/17 (17.6%)	14.13 (3.99–50.06)	14.32 (3.84–53.44)

^*^The model was adjusted for age, Charlson Comorbidity Index, coronary artery disease, American Society of Anesthesiologists (ASA) class, primary tumor diagnosis, neutrophil count, and blood glucose.

OR (95% CI), expressed odds ratios with a 95% confidence anterval (CI).

**Table 3:** Factors associated with postoperative mortality at 30 days.

Characteristics	Univariable		Multivariable	
	OR (95% CI)	*p*-value	OR (95% CI)	*p*-value
Demographics				
Age >65 years	1.43 (1.00–2.05)	0.053	1.39 (0.95–2.04)	0.09
Female	0.79 (0.60–1.03)	0.08	NA	NA
Smoking	0.89 (0.56–1.42)	0.63	NA	NA
Alcohol	1.04 (0.72–1.51)	0.84	NA	NA
Medical history				
Hypertension	0.84 (0.57–1.25)	0.40	NA	NA
Diabetes	1.08 (0.66–1.78)	0.76	NA	NA
Chronic liver disease	0.99 (0.54–1.83)	0.98	NA	NA
Coronary artery disease	2.56 (1.12–5.86)	0.03	1.94 (0.81–4.66)	0.139
ASA class				
I–II	1 [Reference]		1 [Reference]	
III–V	1.44 (1.10–1.88)	0.007	1.23 (0.93–1.61)	0.142
CCI ≥1	2.37 (1.80–3.13)	<0.001	1.22 (0.80–1.85)	0.356
Primary diagnosis				
Meningioma	1 [Reference]		1 [Reference]	
Schwannoma	0.72 (0.28–1.84)	0.49	0.79 (0.31–2.02)	0.62
Craniopharyngioma	4.10 (2.15–7.83)	<0.001	4.54 (2.35–8.79)	<0.001
Glioma WHO grades I–II	1.83 (1.14–2.93)	0.01	1.78 (1.10–2.88)	0.02
Glioma WHO grades III–IV	3.14 (2.12–4.66)	<0.001	2.46 (1.49–4.08)	<0.001
Metastasis	3.26 (1.85–5.74)	<0.001	2.22 (1.16–4.24)	0.02
Other	0.93 (0.55–1.56)	0.78	0.95 (0.56–1.61)	0.84
Systolic blood pressure	0.99 (0.99–1.00)	0.17	NA	NA
Preoperative laboratory				
Platelet	1.00 (1.00–1.00)	0.17	NA	NA
Neutrophil count	1.13 (1.09–1.17)	<0.001	1.08 (1.04–1.12)	<0.001
Blood glucose	1.15 (1.10–1.22)	<0.001	1.12 (1.06–1.19)	<0.001
International normalized ratio	5.99 (1.15–31.12)	0.03	NA	NA
Prothrombin time	1.13 (0.97–1.31)	0.12	NA	NA
Activated partial thromboplastin time	0.99 (0.95–1.02)	0.48	NA	NA
Perioperative medication				
Perioperative transfusion	4.31 (1.32–14.08)	0.02	NA	NA
Preoperative anticoagulant/platelet drugs	1.38 (0.75–2.55)	0.30	NA	NA
Perioperative hematocrit				
Severe anemia	2.80 (1.27–6.14)	0.01	2.24 (0.99–5.06)	0.052
Moderate anemia	1.70 (1.10–2.63)	0.02	1.66 (1.06–2.58)	0.03
Mild anemia	1.04 (0.73–1.47)	0.83	1.12 (0.79–1.60)	0.52
Normal hematocrit	1 [Reference]		1 [Reference]	
Mild polycythemia	1.56 (1.06–2.28)	0.02	1.40 (0.95–2.07)	0.09
Moderate polycythemia	3.20 (1.52–6.71)	0.002	2.81 (1.32–5.99)	0.007
Severe polycythemia	14.13 (3.99–50.06)	<0.001	14.32 (3.84–53.44)	<0.001

ASA, American Society of Anesthesiologists; CCI, Charlson Comorbidity Index; NA, not available.

C-statistics: 0.706.

### Survival analysis

The result of the Kaplan–Meier analysis log-rank test showed that both moderate and severe anemia or polycythemia were associated with worse survival in 1 year compared to normal hematocrit (**Supplementary Figure S1**). In the multivariate Cox regression analysis, the HR was 1.31 (95% CI: 0.97–1.77) for anemia and 1.58 (95% CI: 1.11–2.24) for polycythemia. The HR for severe anemia was 2.23 (95% CI: 1.02–4.85; *p* = 0.044) and 1.70 (95% CI: 1.10–2.63; *p* = 0.016) for moderate anemia; the HR for moderate polycythemia was 2.80 (95% CI: 1.35–5.79; *p* = 0.006) and 10.55 (95% CI: 3.29–33.82; *p* < 0.001) for severe polycythemia (**Supplementary Table S1**).

## Discussion

In this study involving a large retrospective cohort, our results suggest that preoperative anemia was common. Mild anemia was not significantly associated with an increased risk of 30-day mortality, but both moderate and severe anemia were found to be associated with an increased risk, starting at a hematocrit value of less than 0.35. Furthermore, moderate to severe polycythemia was also found to be associated with an increased risk of 30-day mortality, starting at a hematocrit value of more than 0.51.

When comparing the landmark study (2) conducted by Wu et al. on elderly veterans undergoing major noncardiac surgery, different cutoff values were utilized for anemia and polycythemia. The lower cutoff values for anemia (<30.0% in our study versus <39.0% in the study by Wu et al.) indicate a more stringent classification in our study. Conversely, the study by Wu et al. used a broader range for normal hematocrit (39.0%–53.9%) compared to our study’s narrower range of 39.1%–45%. These differences underscore the importance of considering the specific clinical context and the existing evidence when establishing cutoff values for anemia and polycythemia. Collaborative efforts, involving further research and expert consensus, can aid in the establishment of universally applicable cutoff values that align with clinical practice and optimize patient care.

We conducted this study to address key knowledge gaps regarding the association between preoperative anemia polycythemia and postoperative mortality. Prior studies that used the NSQIP database to assess the impact of anemia on patients undergoing elective cranial surgery have reported inconsistent conclusions. One previous study (24) by Alan et al. defined the control group as patients without anemia and matched them with patients with anemia using propensity score matching. The study included a total of 6,576 patients undergoing elective cranial surgery and found that patients with anemia were more likely to have prolonged lengths of stay in the hospital compared to those with no anemia. However, the study did not report any significant differences in postoperative complications or mortality rates between the groups. On the contrary, two previous studies (6, 7) found that preoperative anemia was independently associated with an increased risk of postoperative mortality and morbidity.

The association between polycythemia and postoperative mortality has been studied in various patient populations undergoing different types of surgeries (2, 25–27). For instance, a large study by Wu et al. assessed the effects of preoperative anemia and polycythemia on 30-day postoperative outcomes in elderly veterans undergoing major noncardiac surgery. The study found that even mild degrees of preoperative anemia or polycythemia were associated with an increased risk of 30-day postoperative mortality and cardiac events. The findings of our study are consistent with those of this previous study; however, differences in patient populations, definitions of polycythemia, and outcome measures make direct comparisons challenging. These differences highlight the need for further research to better understand the magnitude and consistency of the association between polycythemia and postoperative mortality across various patient populations and surgical procedures.

In comparison to previous studies, our study provides novel insights into the relationship between preoperative anemia, polycythemia, and postoperative mortality. Our study defined the control group as patients without anemia or polycythemia. We found that not only moderate to severe anemia but also polycythemia is independently associated with increased postoperative mortality in patients undergoing craniotomy for brain tumor resection. We also found that the risk of mortality increases with the severity of both conditions. Lastly, our study assessed the impact of mild anemia and longer-term mortality. Our study suggests that managing these conditions proactively before performing the surgery may reduce the risk of adverse outcomes.

To examine the impact of comorbidities that are typically present in patients with abnormal hematocrit values, we calculated the Charlson Comorbidity Index score. Even after adjusting for this important confounder, our results remained robust. Therefore, it is possible that the potential mechanism behind preoperative anemia and polycythemia increasing the risk of postoperative mortality is related to abnormal hematocrit values rather than their comorbid disease burden.

Numerous limitations require consideration in this study. The retrospective design poses challenges in confirming direct causality and is susceptible to predisposition from unobserved variables like oxygen saturation and chronic hypoxemia, such as obstructive sleep apnea, heart disease, or obesity hypoventilation syndrome, which might have influenced our findings (26, 28, 29). Second, our hospital data could not capture the reason for anemia or polycythemia. Future studies should explore the reason for anemia or polycythemia and when treatment should be taken. Lastly, the lack of accepted criteria for defining the severity of anemia and polycythemia hinders the generalizability of these findings.

Our study has important implications for clinical practice. Our findings underscore the importance of identifying and managing abnormal hematocrit values before craniotomy surgery. Specifically, preoperative management of hematocrit values for patients with moderate to severe anemia or polycythemia may reduce the risk of postoperative mortality. Future research should focus on identifying the mechanisms underlying the observed associations between abnormal hematocrit values and postoperative mortality and developing effective interventions to manage hematocrit values in patients undergoing craniotomy.

## Conclusions

There exists a U-shaped relationship between hematocrit and postoperative mortality in patients who underwent craniotomy for brain tumor resection. Moderate to severe anemia and polycythemia are associated with increased postoperative mortality in patients undergoing craniotomy for brain tumor resection. These findings underscore the importance of identifying and managing abnormal hematocrit values before craniotomy surgery.

## Data availability statement

The original contributions presented in the study are included in the article/**Supplementary Material**. Further inquiries can be directed to the corresponding author.

## Ethics statement

The studies involving humans were approved by the ethics committee of West China Hospital (No. 2022-705). The studies were conducted in accordance with the local legislation and institutional requirements. Written informed consent for participation was not required from the participants or the participants’ legal guardians/next of kin in accordance with the national legislation and institutional requirements.

## Author contributions

Study concept: FF. Design: all authors. Acquisition, analysis, or interpretation of data: YX, YZ, XC, LJ, YT, JH, MH, LC, PH, TL, LP, WC, YH, CY, and FF. Statistical analysis: YX and YZ. Drafting of the manuscript: YZ and YX. Critical revision of the manuscript for important intellectual content: All authors. All authors contributed to the article and approved the submitted version.
