# Supplementary material for: Preoperative hematocrit levels and postoperative mortality in patients undergoing craniotomy for brain tumors
Source: Front Oncol. 2023 Oct 17;13:1246220. doi: 10.3389/fonc.2023.1246220 (PMC10616849; doi:10.3389/fonc.2023.1246220)
Supplement: Supplementary file 1 [file DataSheet_1.docx]

**Supplementary Materials**

**Table S1. Cox Proportional Hazards Model for 30-day Mortality, stratified by Hematocrit.**

HCT： Hematocrit

**Figure S1 Kaplan-Meier Estimates for Overall Survival for Patients Undergoing Elective Craniotomy for A Brain Tumor**

(A): Overall Survival for Patients with anemia. (B): Overall Survival for Patients with polycythemia

HCT： Hematocrit

**Table S1. Cox Proportional Hazards Model for 30-day Mortality, stratified by Hematocrit.**

| Characteristics | Hematocrit | Events/Total,  n (%) | Unadjusted  HR (95% CI) | Logistic Regression  Adjustment  HR (95% CI) * |
| --- | --- | --- | --- | --- |
| Severe Anemia | ≤0.3 | 7/172(4.1%) | 2.76(1.28- 5.96) | 2.23(1.02- 4.85) |
| Moderate Anemia | 0.301-0.35 | 27/1075(2.5%) | 1.69(1.10- 2.60) | 1.70(1.10- 2.63) |
| Mild Anemia | 0.351-0.39 | 51/3286(1.6%) | 1.04(0.74- 1.47) | 1.13(0.80- 1.60) |
| Normal Hematocrit | 0.391-0.45 | 86/5756(1.5%) | 1(Reference) | 1(Reference) |
| Mild Polycythemia | 0.451-0.5 | 39/1691(2.3%) | 1.55(1.06- 2.26) | 1.39(0.95- 2.04) |
| Moderate Polycythemia | 0.501-0.55 | 8/173(4.6%) | 3.13(1.51- 6.45) | 2.80(1.35- 5.79) |
| Severe Polycythemia | >0.55 | 3/17(17.6%) | 12.88(4.07-40.71) | 10.55(3.29-33.82) |


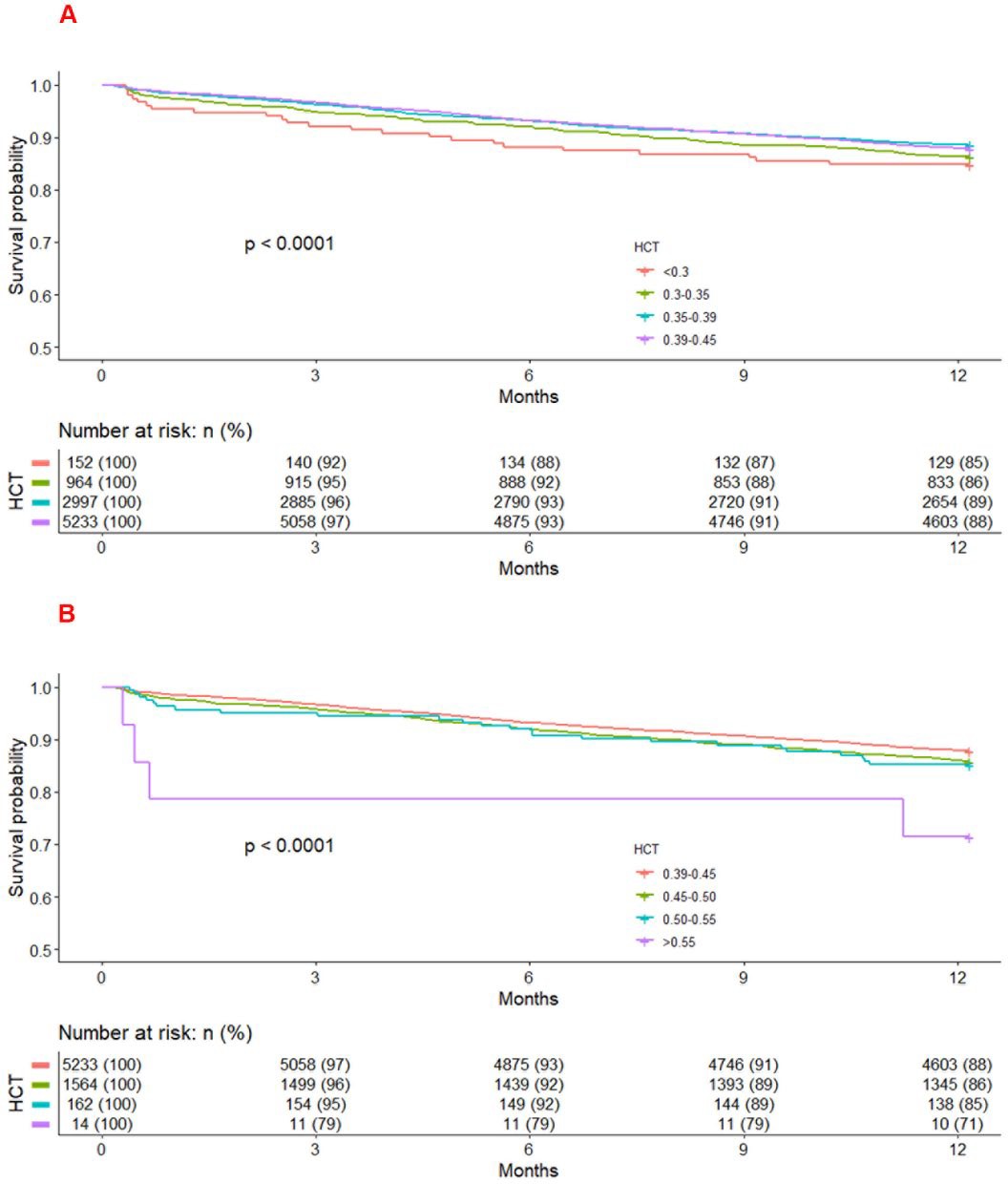


**Figure S1 Kaplan-Meier Estimates for Overall Survival for Patients Undergoing Elective Craniotomy for A Brain Tumor**

(A): Overall Survival for Patients with anemia. (B): Overall Survival for Patients with polycythemia

HCT: Hematocrit
